# Black soldier fly: a new model for bioremediation of antibiotic pollutants

**DOI:** 10.1093/nsr/nwaf043

**Published:** 2025-02-10

**Authors:** Xia Xu, Yongping Huang, Xingyu Luo

**Affiliations:** School of Environmental Science and Engineering, Shanghai Jiao Tong University, China; Institute of Sericulture and Tea, Zhejiang Academy of Agricultural Sciences, China; School of Environmental Science and Engineering, Shanghai Jiao Tong University, China; State Key Laboratory of Microbial Metabolism, School of Life Sciences and Biotechnology, Shanghai Jiao Tong University, China; School of Environmental Science and Engineering, Shanghai Jiao Tong University, China

Insects, as a dominant portion of animal biomass, are present in all terrestrial ecosystems and perform important ecological roles such as pollination, herbivory, carnivory and decomposition. Insects play a crucial role in decomposing animal feces, carcasses and plant-derived organic matter, prompting exploration into insects as bio-agents for nutrient recycling from organic waste in households, industries and agriculture [1]. Currently, insects are also being further explored for the treatment of organic waste containing pollutants such as pharmaceuticals and pesticides [2–4].

Antibiotics are common pollutants in organic waste. Antibiotic pollutants in organic waste can reach the environment and threaten human health by promoting the emergence and spread of pathogenic resistant bacteria through mutations or horizontal gene transfers [5]. Two types of organic waste often containing residual antibiotics are animal manure and antibiotic fermentation residues (AFR). In China alone, approximately 3 billion tons of animal manure are produced annually [6]. Two million tons of AFR, a by-product of microbial antibiotic production containing high levels of residual antibiotics and organic matter, are generated annually in China [7]. The sheer volume of the two types of waste presents a formidable challenge for effective management. Current waste management strategies include incineration, aerobic composting and hydrothermal treatment; however, these approaches either incur significant costs related to equipment and energy or fail to recycle essential macro-nutrients, resulting in significant carbon emissions [3,8]. Therefore, we urgently need to seek new methods for the management of such waste.

## BLACK SOLDIER FLIES ARE A PROMISING BIO-AGENT TO TREAT ORGANIC WASTE

**Figure 1.**
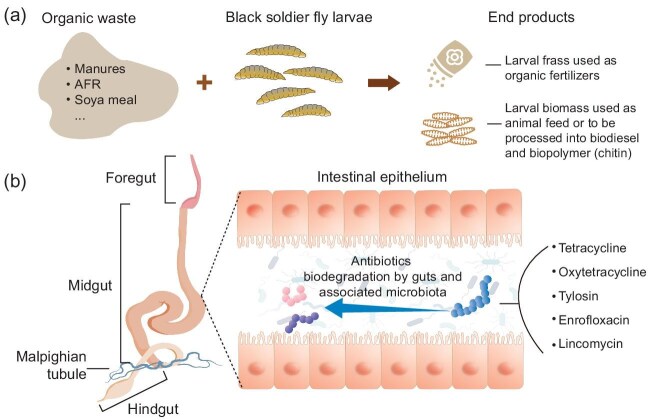
(a) End products of black soldier fly larvae (BSFL)-mediated bioconversion of organic waste. (b) Diagram of BSFL gut structure and the biodegradation of antibiotics by BSFL gut configuration and the associated microbiota.

Recently, the black soldier fly *Hermetia illucens* L. (Diptera: Stratiomyidae) has garnered considerable attention as an innovative invertebrate bio-agent due to its exceptional ability to process organic waste and the high value of the end products (Fig. 1a). Black soldier fly larvae (BSFL) can consume a diverse array of substrates, including kitchen waste, agricultural residues and animal manure. The larval biomass generated through bioconversion of organic waste is rich in proteins, lipids and chitin [9]. The treatment residue (frass), which contains a mixture of larval excrements and molted cuticles, can be used as organic fertilizer, promoting crop growth. Furthermore, BSFL have been shown to mitigate greenhouse gas emissions during waste treatment, with roughly 30% of the carbon and 55% of the nitrogen from the waste retained in the larval biomass [10]. The bioconversion of organic waste by BSFL offers a sustainable solution to the global challenges of waste management and the increasing demand for alternative protein sources (Fig. 1a) [9–11]. Consequently, BSFL farming facilities have been established worldwide, with the end products increasingly used for animal feed, organic fertilizers, biodiesel and biopolymers (chitin) in recent years [12].

The microbiota residing in the gut of BSFL significantly contribute to the capacity of larvae to efficiently degrade organic matter [13] and various xenobiotics, including pesticides, mycotoxins and antibiotics [11]. The bacterial community found in both BSFL guts and frass are highly diverse, encompassing hundreds of distinct bacterial species, with Proteobacteria and Firmicutes being the top two dominant phyla involved in the BSFL-mediated bioconversion of animal manure and AFR [3,14]. These phyla are often associated with xenobiotic metabolism. Moreover, BSFL possess a compartmentalized digestive tract with α-amylase, lipase, endopeptidase, exopeptidase and lysozyme activities, enabling larvae to consume substantial quantities of food [15]. The larval gut comprises the foregut, midgut and hindgut, with the midgut being the largest compartment (Fig. 1b). The midgut can be further segmented based on pH values into three distinct regions: the anterior midgut (acidic, pH 6.0), middle midgut (strongly acidic, pH 2.0) and posterior midgut (alkaline, pH 8.5) [15], and the wide range of pH levels within insect midguts may play a pivotal role regulating digestive enzyme activity, nutrient solubility and xenobiotic degradation.

## BSFL AND THE ASSOCIATED MICROBIOTA CAN DEGRADE A DIVERSE ARRAY OF ANTIBIOTICS

BSFL display a remarkable ability to degrade a broad spectrum of antibiotics in various organic substrates. For instance, BSFL can effectively degrade up to 97% of tetracycline in spiked wheat bran within 12 days [16]. Similarly, the larvae can degrade over 60% of oxytetracycline in fermentation residues mixed with soya meal within 7 days [4]. When consuming swine manure with tylosin and enrofloxacin, the larvae can achieve 82.9% and 80.9% degradation of respective antibiotics within 12 days [17]. Additionally, the larvae exhibit a potent ability to degrade lincomycin, degrading up to 84.9% of the antibiotic within 12 days when fed with lincomycin fermentation residue mixed with wheat bran [3]. The variability in degradation efficiency may arise from the differences in antibiotic classification and experimental conditions across those studies. Notably, none of the antibiotics mentioned above were detected in the larval body, except for enrofloxacin, which showed decreasing levels from larvae to pupae [17]. This indicates that the presence or absence of residual antibiotics in the larval body may depend on factors such as the type of antibiotic, exposure concentration and duration of exposure, highlighting the necessity for scrutiny of larvae generated from antibiotic remediation before their use. Collectively, these findings underscore the potential of BSFL as an effective bioremediation tool for antibiotic-contaminated organic waste.

Antibiotic resistance genes (ARGs), which are typically harbored on mobile genetic elements and responsible for bacterial resistance to antibiotics, have also been identified in the context of larval guts [3,4,16,17]. Research on different antibiotics reveals a consistent pattern: a significant increase in the abundance of corresponding ARGs in larval guts fed with a particular antibiotic, followed by the decrease of certain resistance genes as the antibiotic degrades, indicating microbial adaptation to the antibiotics [3,16,17]. Fortunately, BSFL are not supposed to be released into the open-air environment during insect farming, and the post-harvest processing of larvae often involves blanching, oven-heating and drying [18], which may eliminate the associated microbes, thereby significantly mitigating the potential environmental risks posed by the presence of ARGs in larval guts.

The mechanisms underlying the degradation of antibiotics during the bioconversion of organic waste by BSFL have been explored (Fig. 1b), revealing that both the larvae and their associated microbiota play crucial roles [3,16,17]. Several antibiotic-degrading bacterial strains have been isolated from the larval guts, confirming the microbiota's contribution. By establishing a sterile larval rearing condition, the distinct contributions of the larvae and their microbiota to degradation can be observed: the larval gut microbiota can double the degradation rates of tetracycline [16], and accounts for 47.5% and 72.1% of the total tylosin and enrofloxacin degradation [17], and 22.0% of the total lincomycin degradation [3]. Furthermore, these studies have also confirmed that the larvae themselves possess the ability to degrade antibiotics, likely facilitated by their gut configuration, which includes digestive enzymes and a broad pH range [15]. The larvae can also synergize with symbiotic antibiotic-degrading bacteria to enhance antibiotic degradation further [3,16,17]. However, few studies have investigated the molecular mechanisms underlying the biodegradation, and the cross-kingdom synergy involved. Despite the fact that ARGs related to microbial degradation can be detected during the process, no direct evidence is provided in those studies to prove their role in biodegradation [3,16,17].

## PERSPECTIVES ON BSFL-MEDIATED ANTIBIOTIC REMEDIATION

The potential of BSFL to remediate antibiotic pollutants from organic waste is promising not only due to their exceptional capacity to degrade residual antibiotics but also their ability to assimilate residual nutrients of the waste into biomass, thus reducing carbon emissions. Feeding swine manure with residual tylosin and enrofloxacin alone is sufficient to support larval growth [17]. And a combination of AFR with other organic waste such as wheat bran and soya meal, can also sustain the complete life cycle of the insects [3,4]. Overall, this approach generates valuable biomass while alleviating the societal burden associated with managing harmful organic waste, thus potentially outperforming alternative strategies for managing antibiotic pollutants.

One concern hindering the large-scale application of the technology is the limited understanding of the environmental toxicity of degradation intermediates. Few studies have chemically characterized the degradation pathway of antibiotic molecules during BSFL bioconversion, which is an essential step prior to assessing toxicity and further ecological impact. Another obstacle is the limited capability to degrade certain types of antibiotics, exemplified by the treatment oxytetracycline fermentation residues, where approximately 40% oxytetracycline remains undegraded [4]. While modifying parameters, such as the number of BSFL inoculated, the bioconversion temperature, or microbiota composition, may enhance degradation, understanding the molecular mechanisms of host–microbe synergy in degradation could lead to more effective outcomes. So far, a limited number of studies have focused on the antibiotic-degrading genes or pathways within the diverse microbial pool in larval guts and frass, or the insect genome, which has undergone a substantial expansion in functional modules related to the detoxification of xenobiotics [14]. Investigating whether a shared biodegradation pathway exists between the host and gut microbiota is also an intriguing area for further research. Future studies aimed at identifying the genes or pathways involved in biodegradation could provide a list of candidate functional elements for genetic integration of antibiotic-degrading capabilities into the BSFL genome and larvae-associated microbiome, thereby enhancing biodegradation efficacy.
